# Single-cell RNA sequencing reveals immune regulatory mechanisms and molecular therapeutic strategies in the microenvironment of multiple myeloma

**DOI:** 10.1097/JS9.0000000000003306

**Published:** 2025-09-03

**Authors:** Qiang Su, Kehan Long, MoZiLi Adu, Meijun Jiang, Qiaochu Li, Xufeng Wan, Jian Cao, Yan Yue, Shuoyuan Li, Zhendong Ying, Qibin Liu, Chaoqun You, Zhuang Zhang, Duan Wang

**Affiliations:** a Orthopedic Research Institute and Department of Orthopedics, West China Hospital, Sichuan University, Chengdu 610041, China; b School of Clinical Medicine, Shandong Second Medical University, Weifang, Shandong, China; c Research Center for Traditional Chinese Medicine Resources and Ethnic Minority Medicine, Jiangxi University of Chinese Medicine, Nanchang, China; d Department of Hand and Podiatric Surgery, Orthopedics Center, The First Hospital of Jilin University, Changchun, People’s Republic of China; e Department of Orthopedics, the First Affiliated Hospital, Chongqing Medical University, Chongqing, China; f Department of Orthopedics, School of Clinical Medicine, Shandong University of Traditional Chinese Medicine, Shandong Qianfoshan Hospital, Jinan, China; g Affiliated Hospital of Shandong Second Medical University, Jinan, China; h Musculoskeletal Tumor Center, Beijing Key Laboratory of Musculoskeletal Tumor, Peking University People’s Hospital, Beijing, China

**Keywords:** immune modulation, Mendelian randomization, molecular docking, multiple myeloma, scRNA-seq, therapeutic targets

## Abstract

**Background:**

Multiple myeloma (MM) is a malignancy marked by uncontrolled plasma cell proliferation, immune evasion, and drug resistance. Despite advances in treatment, the disease remains incurable due to relapses and drug resistance. This study aims to investigate the molecular and cellular interactions within the myeloma microenvironment using single-cell RNA sequencing (scRNA-seq), Mendelian randomization (MR), and pathway analysis to uncover therapeutic targets.

**Methods:**

We used scRNA-seq to analyze tumor, immune, and stromal cell interactions in MM. Data were processed using Seurat for clustering, dimensionality reduction, and cell-type annotation. Gene Set Variation Analysis (GSVA) and cell–cell interaction analysis were performed to identify signaling pathways involved in disease progression. Two-sample MR was applied to explore causal relationships between genetic variants and gene expression. Finally, molecular docking simulations were used to identify potential small molecule modulators of key proteins involved in MM.

**Results:**

We identified significant immune-related signaling pathways, including PI3K-AKT-mTOR, WNT-β-catenin, and TGF-β, upregulated in immune cells within the MM microenvironment. Genes such as HLA-C, CTSS, and LRRFIP1 showed positive causal relationships with MM, while SHISA5 and ISG15 exhibited protective roles. Cell communication analysis revealed key ligand–receptor interactions between immune and tumor cells. Molecular docking identified promising small molecules like actein and aflatoxin B1 targeting ISG15 and TAGLN2.

**Conclusions:**

This study reveals key genetic drivers and immune modulation mechanisms in MM. Targeting immune-related pathways, such as PI3K-AKT-mTOR and WNT-β-catenin, and small molecules targeting ISG15 and TAGLN2 could offer new therapeutic strategies.

## Introduction

Multiple myeloma (MM) is a serious blood cancer in which plasma cells begin to proliferate uncontrollably in the bone marrow, leading to severe health complications and, unfortunately, high mortality rates^[1,2]^. Clinically, the disease is marked by the presence of abnormal monoclonal proteins, or M-proteins, in the blood or urine[3]. Alongside this, patients often face a range of debilitating issues like anemia, bone damage, kidney failure, and a weakened immune system[4]. Even though modern treatments – such as proteasome inhibitors, immunomodulatory drugs, and monoclonal antibodies – have made strides, MM remains incurable^[5,6]^. A significant challenge lies in the frequent relapses caused by the development of drug-resistant strains[7]. To tackle this issue, it is crucial to explore the molecular mechanisms behind disease progression, the complexity of cellular diversity, and the tumor environment itself[8]. This deeper understanding could guide the development of more personalized treatments that offer improved outcomes for patients.

Recent breakthroughs in genomic technologies, especially single-cell RNA sequencing (scRNA-seq), have opened new doors to understanding the vast diversity of cells within the myeloma microenvironment[9]. This technique allows researchers to investigate gene expression at a granular level, revealing the intricate mix of tumor, immune, and stromal cells – details often missed in traditional bulk tissue sequencing[10]. These advancements provide a more comprehensive picture of the disease, identifying the key factors that drive disease progression, immune evasion, and resistance to therapy. Gaining a better understanding of how different cell types interact within the myeloma environment, including tumor cells, immune cells, and stromal elements, is crucial for deciphering the complexities of MM and for developing strategies to target specific biological pathways[11]. One of the toughest challenges in treating MM lies in the constantly shifting nature of the tumor microenvironment. The immune system plays a critical role in either supporting or obstructing MM’s progression. Interactions between immune cells – such as T cells, NK cells, and monocytes – and myeloma cells can influence the tumor’s ability to avoid immune detection^[12,13]^. The immune landscape in MM is often characterized by a suppression of immune responses, where immune cells struggle to effectively respond to the myeloma cells[14]. Understanding the mechanisms behind this immune dysfunction is pivotal in designing immunotherapies that can reactivate the immune system to better fight the cancer.

This study aims to elucidate the cellular architecture and intercellular signaling dynamics within the myeloma microenvironment using scRNA-seq. By resolving immune and stromal cell heterogeneity, we identify key molecular signatures and transcriptional states that potentially drive disease progression and resistance to therapy. We further employ gene set variation analysis (GSVA) and cell–cell communication modeling to characterize the signaling pathways and ligand–receptor interactions that mediate immunosuppression and tumor support in MM^[15,16]^. To establish causal links between gene expression and disease susceptibility, we integrate eQTL data with genome-wide association study (GWAS) results through two-sample Mendelian randomization (MR)[17]. This approach allows us to prioritize genes whose expression alterations are likely to influence MM pathogenesis, distinguishing causal drivers from correlative markers. By connecting genetically regulated expression with single-cell transcriptional patterns, we offer a novel framework to interpret functional gene-disease relationships in the context of the tumor microenvironment.

Pathway enrichment analyses further delineate the biological processes enriched among pathogenic cell states, highlighting pathways such as PI3K-AKT-mTOR, WNT-β-catenin, and TGF-β that are aberrantly activated in MM. These pathways are known to govern cellular survival, proliferation, and immune escape[18]. Targeting these dysregulated signaling axes may offer opportunities to restore immune function and inhibit tumor progression. Moreover, mapping the intercellular ligand–receptor interactions provides insights into how immune dysfunction is orchestrated through specific communication circuits, revealing actionable targets for immunotherapeutic intervention[19]. To translate these insights into therapeutic potential, we performed molecular docking simulations to screen small molecules against candidate proteins identified through MR[20]. By modeling the binding interactions between compounds and their targets, we identify structurally viable molecules – such as those modulating ISG15 and TAGLN2 – with the potential to alter immune regulatory mechanisms in MM.

In summary, our integrative approach combines single-cell transcriptomics, genetic causal inference, and virtual screening to uncover molecular determinants and therapeutic vulnerabilities in MM. This strategy not only enhances mechanistic understanding of immune evasion but also facilitates the identification of novel therapeutic candidates tailored to the complex landscape of the myeloma microenvironment.

**HIGHLIGHTS**

- Single-cell RNA sequencing (scRNA-seq) and Mendelian randomization (MR) were integrated to dissect the molecular interactions in the multiple myeloma (MM) microenvironment.
- Gene Set Variation Analysis (GSVA) uncovers immune escape mechanisms driven by key pathways, including PI3K-AKT-mTOR and WNT-β-catenin.
- Key genes such as *HLA-C, CTSS*, and *LRRFIP1* were identified as potential risk factors, while *SHISA5* and *ISG15* exhibited protective roles in MM.
- Ligand–receptor interaction analysis revealed critical crosstalk between tumor and immune cells, influencing disease progression.
- Molecular docking simulations identified actein and aflatoxin B1 as potential small-molecule modulators targeting *ISG15* and *TAGLN2*, offering novel therapeutic strategies for MM.

Given the increasing integration of artificial intelligence (AI) tools in biomedical research, this study adheres to the TITAN (Transparency In The reporting of Artificial INtelligence) Guidelines 2025 to promote methodological transparency and reproducibility[21].

## Methods

### Study design

This study utilized scRNA-seq to investigate the cellular characteristics and interaction networks within the context of multiple myeloma. Through the integration of single-cell data, we performed cross-processing of marker genes for each cell type to identify genes that are commonly expressed across different cell clusters. The intersected genes were subsequently examined using two-sample MR to assess their causal relationships with multiple myeloma. This MR analysis identified key genes that may drive the pathogenesis of multiple myeloma, which were further explored for target prediction and molecular docking to uncover potential protein interactions and prioritize promising therapeutic targets.

**Figure 1.**
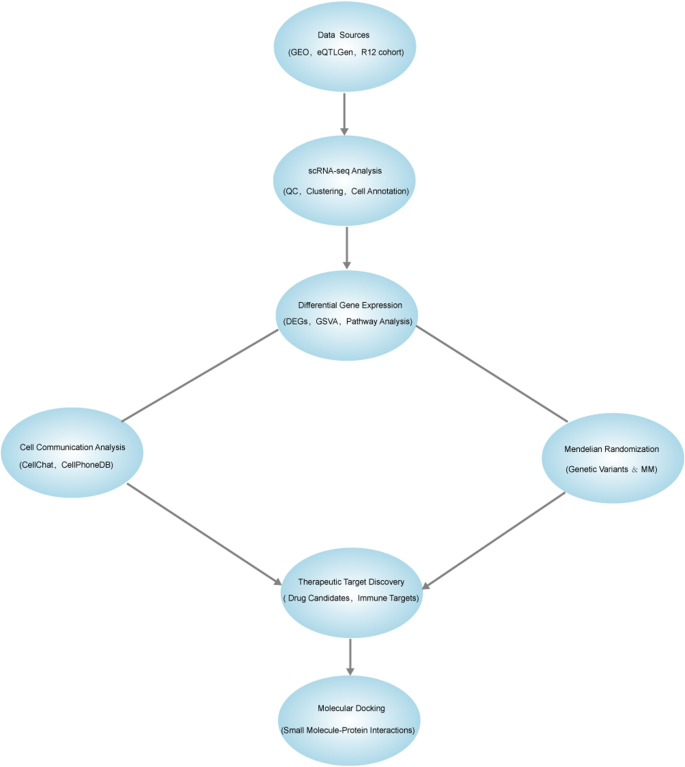
Study workflow. An integrated flowchart outlining the study design and methodology. Key steps include single-cell RNA sequencing (scRNA-seq) data preprocessing, differential gene identification, Mendelian randomization analysis, cell–cell communication modeling, and molecular docking for therapeutic exploration.

In parallel, GSVA and cell–cell interaction analysis were employed to explore the underlying signaling pathways and cellular communications contributing to multiple myeloma’s pathogenesis. These analyses enabled the identification of key molecular pathways involved in the disease, offering insights into therapeutic targets and potential treatment strategies. Specifically, Fig. 1 provides an overview of the methodological flow and analytical approach used in the study, guiding the process from data collection to target prediction and pathway analysis.

### Data sources

All cis-eQTL data used in this study were sourced from the eQTLGen database (https://www.eqtlgen.org/), which provides comprehensive genetic information about expression quantitative trait loci across various populations^[22,23]^. This database is a key resource for identifying genetic variants that regulate gene expression, offering insights into the genetic underpinnings of diseases like multiple myeloma.

The multiple myeloma data for this study were obtained from FinnGen R12, which includes 1608 European Antarctic cases of multiple myeloma and 378,739 European Antarctic control cases[24]. This large dataset is instrumental in understanding the genetic associations with multiple myeloma and provides a foundation for further analyses of gene-disease relationships.

Additionally, the scRNA-seq data for multiple myeloma patients and their paired adjacent tissues were retrieved from Gene Expression Omnibus (GEO) under accession number GSE271107^15^. This dataset includes data from healthy donors, MM, and smoldering multiple myeloma (SMM) samples. These data were selected to explore the differential gene expression and cellular heterogeneity within the multiple myeloma microenvironment, comparing it to normal tissue and providing insights into the progression from healthy states to more aggressive disease forms.

### Single cell analysis

#### Data preprocessing and Seurat object creation

Data preprocessing was performed using the Seurat package in R, which was used to process the scRNA-seq data and create Seurat objects. First, the expression matrices from the scRNA-seq data were loaded into R, and the data from each sample were integrated into a single Seurat object for further analysis. To ensure the quality of the data, low-quality cells were filtered out based on predefined criteria. Quality control (QC) was performed to ensure reliability of the single-cell RNA-seq data. Three key metrics were used to assess cell quality: RNA feature count (nFeature_RNA), total RNA count (nCount_RNA), and mitochondrial gene expression ratio (percent.mt). Cells were excluded if they met any of the following criteria: percent.mt <25%, nCount_RNA <45 000, or nFeature_RNA <6000. These thresholds helped remove low-quality cells and minimize technical artifacts, ensuring that downstream analyses reflect true biological variation^[25,26]^. These filtering steps helped to remove cells that were likely to be of poor quality or irrelevant to the study.

#### Dimensionality reduction, clustering, and annotation

To reduce the dimensionality of the single-cell RNA-seq data and visualize the high-dimensional data in a two-dimensional space, the UMAP (Uniform Manifold Approximation and Projection) algorithm was applied^[27,28]^. This technique effectively captures the global structure of the data while preserving local relationships between cells. After dimensionality reduction, cell clustering was performed using the FindClusters function in Seurat. The resolution parameter was adjusted to optimize the clustering results, allowing for the identification of distinct cell populations[29].

For cell type annotation, the SingleR package in R was used. SingleR is an automated tool that assigns cell types to individual cells based on a reference transcriptome dataset[30]. Cell type annotation was performed using the SingleR package (v2.6.0) with the BlueprintEncodeData reference from the celldex package. This reference dataset contains curated gene expression profiles of sorted immune and stromal cell populations. To improve computational efficiency and ensure reproducibility, the reference object was downloaded once and saved locally as ref BlueprintEncodeData.RData.

The input test data consisted of the log-normalized gene expression matrix (scRNA@assays$RNA@data), and initial cluster identities were defined by the Seurat object’s active.ident slot. Annotation was conducted in cluster mode, specifying labels = ref$label.main to obtain major lineage assignments. To enhance annotation accuracy, we used the pruned.labels output from SingleR, which filters out ambiguous or low-confidence predictions. These refined cluster-level labels were subsequently used to update the cluster identities within the Seurat object via RenameIdents().

Of note, although erythrocytes and hematopoietic stem cells (HSCs) were annotated through this automated process, they were not considered a biological focus of the present study and were excluded from downstream analyses, including pathway, cell–cell communication, and causal inference analyses.

#### Candidate gene analysis

To identify key genes involved in the pathogenesis of multiple myeloma, we performed intersection processing of marker genes across various cell types. We cross-referenced these genes with existing literature, excluding previously studied genes. The expression patterns of the remaining candidate genes were visualized using the EigenPlot and VlnPlot functions in Seurat, providing insight into how these genes are expressed across different cell clusters[31]. This approach helped to prioritize novel genes that could serve as potential therapeutic targets.

#### Pathway enrichment analysis

To explore the functional pathways associated with the identified candidate genes, pathway enrichment analysis was performed using the GSVA package. We downloaded the latest GMT format gene set files from the GSEA-msigdb database, which provides a collection of gene sets for various biological pathways[32]. This analysis evaluated the activity levels of key pathways across different cell types, helping to reveal the underlying biological processes that might be driving the progression of multiple myeloma.

#### Cellular communication analysis

Cell–cell communication analysis was performed using two widely adopted tools: CellChat (v2.1.2) and CellPhoneDB (v4.1.0). For CellChat, we used the CellChatDB.human database and restricted the analysis to “Secreted Signaling” pathways. Overexpressed genes and ligand–receptor interactions were identified using the identifyOverExpressedGenes() and identifyOverExpressedInteractions() functions. Ligand–receptor pairs were projected onto the PPI network using projectData() with the PPI.human object. Communication probability and pathway-level inference were computed using computeCommunProb() and computeCommunProbPathway(), respectively, with default parameters. Aggregated interaction networks were generated using aggregateNet(), and signaling roles of each cell type were inferred by computing centrality metrics via netAnalysis_computeCentrality(). Visualization of cell type-specific signaling contributions was carried out using netAnalysis_signalingRole_network(). For CellPhoneDB, we used the standard pipeline with default statistical parameters. Normalized gene expression data and cell annotations were input into the software, and interaction significance was evaluated using the built-in permutation test (n = 1000) to identify enriched ligand–receptor pairs between cell types.

#### Software and visualization

All analyses were conducted in the R environment (v4.4.1), ensuring reproducibility and consistency. A comprehensive suite of R packages was employed across different stages of the study:

Single-cell data processing and annotation: Seurat (v5.1.0) was used for data normalization, dimensionality reduction, clustering, and visualization. Cell type annotation was performed using SingleR (v2.6.0). Cell–cell communication analysis: Conducted using CellChat (v2.1.2) with the CellChatDB.human database and CellPhoneDB (v4.1.0), applying default settings for ligand–receptor inference and interaction significance testing. Mendelian randomization analysis: TwoSampleMR (v0.6.14), MRPRESSO (v1.0), and TSMRhelper (v0.0.9) were used for causal effect estimation, pleiotropy testing, and sensitivity analysis visualization. Pathway activity and enrichment analysis: GSVA (v1.52.3) was used with hallmark gene sets from the MSigDB database. Visualization tools: ggplot2 (v3.5.1) was used for general plotting; ggpubr (v0.6.0) and ggsci (v3.2.0) for publication-ready layouts and color themes; plotly (v4.10.4) for interactive visualization; ggview (v0.1.0) for preview rendering; and forestploter (v1.1.2) for forest plots. Data manipulation and utilities: dplyr (v1.1.4), data.table (v1.15.4), and stringr (v1.5.1) were used for data wrangling. Base R packages such as parallel, grid, and grDevices supported computational and graphical tasks.

This detailed specification of software and package versions ensures transparency, reproducibility, and methodological rigor throughout all analytical procedures.

### MR analysis

To investigate the causal relationship between genetic variations and gene expression levels in multiple myeloma, we first obtained genetic variations significantly correlated with gene levels from genome-wide association studies (GWAS). These data were used to explore the role of cis-eQTLs (expression quantitative trait loci), which are genetic variants located near the genes they regulate[33]. To ensure the validity of cis-eQTLs as instrumental variables (IVs) in the MR analysis, we applied a series of stringent filtering and validation procedures. First, SNPs were required to reach genome-wide significance (*P* < 5 × 10^−8^) and be located within ±1 Mb of the transcription start site of the corresponding gene. To ensure independence among variants, linkage disequilibrium pruning was performed with a threshold of R^2^ <0.01. Instrument strength was evaluated using F-statistics, with F >10 indicating sufficient strength to mitigate weak instrument bias. Furthermore, we performed the Steiger directionality test to confirm the correct causal direction from gene expression to disease risk. These validation steps collectively enhance the reliability of the instrumental variables used in the MR framework.

To perform MR analysis, the TwoSampleMR software package in R was used. This package enables the evaluation of causal relationships between genetic variants and diseases by leveraging genetic data. In the case of cis-eQTLs with only a single SNP (single nucleotide polymorphism), the Wald ratio method was used as the standard[34]. The Wald ratio is a simple method to estimate causal effects when there is only one SNP involved, offering a straightforward calculation of the causal relationship between the genetic variant and gene expression.

For cis-eQTLs with multiple SNPs, the results of inverse variance weighting (IVW) were used as the preferred method^[35,36]^. IVW combines the effects of multiple genetic variants and provides a more robust estimate of causality when multiple SNPs are associated with the gene expression. The IVW method aggregates the individual effects of each SNP while accounting for their respective variances, yielding a more reliable estimate of the causal relationship.

To further validate the findings and rule out the possibility of reverse causality, the Steiger test was employed. This test helps detect whether the observed associations between SNPs and gene expression could be due to reverse causality, where gene expression influences the SNP rather than the other way around[37]. The test compares the direction of the association between SNPs and gene expression to determine if the causal inference holds.

### Molecular docking

The Comparative Toxicogenomics Database (CTD, https://ctdbase.org/) is a public resource that facilitates the understanding of how environmental exposures, including chemicals, influence human health[38]. In this study, CTD was used to identify small molecules that modulate the expression of genes causally associated with multiple myeloma. For genes positively associated with disease risk, we selected compounds known to downregulate their expression; conversely, for protective genes, we selected upregulating molecules[39].

Structural data for selected small molecules were downloaded from PubChem and converted from SDF to mol2 format using Open Babel[40]. Receptor proteins were prepared by removing water molecules and ligands in PyMOL, followed by hydrogenation and charge assignment using AutoDock Tools[41]. Both ligands and receptors were converted to PDBQT format for docking via AutoDock Vina[42].

Docking results were visualized in PyMOL to examine ligand–receptor binding orientations and interaction sites^[43,44]^. Protein sequence and domain information were retrieved from UniProt (https://www.uniprot.org/) to facilitate biological interpretation of binding sites[45]. These analyses helped identify potential therapeutic targets and assess how candidate compounds may influence protein function in the context of multiple myeloma.

### AI-assisted language editing disclosure

During the preparation of this manuscript, the authors used ChatGPT (OpenAI, GPT-4.0) to assist with language refinement, including grammar correction, clarity improvement, and editorial polishing. This tool was employed exclusively for language editing purposes, and no generative AI was used for data analysis, figure creation, or scientific interpretation. All AI-generated suggestions were manually reviewed, verified, and edited by the authors to ensure accuracy, appropriateness, and consistency with the intended scientific message. The authors take full responsibility for the integrity and accuracy of the final content. This disclosure is made in accordance with the TITAN Guidelines 2025, to promote transparency and reproducibility in scientific reporting[21].

## Results

### Quality control and sample characteristics

**Figure 2.**
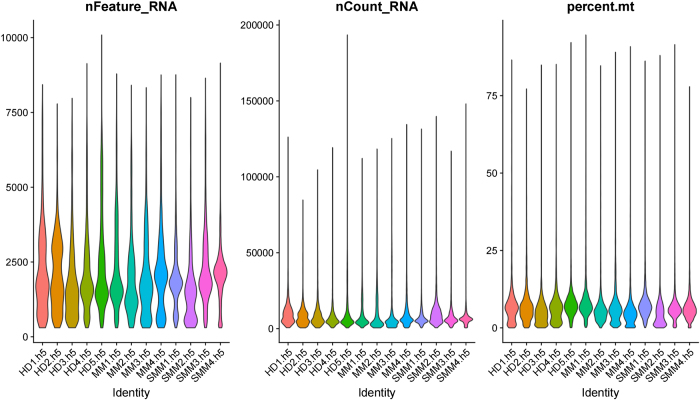
Violin plots of single-cell quality control metrics across all samples. (Left) number of detected genes per cell (nFeature_RNA); (Middle) total RNA molecule counts (nCount_RNA); (Right) proportion of mitochondrial gene expression (percent.mt). Each sample corresponds to a unique color-coded distribution. Cells with percent.mt <25% or nCount_RNA <45 000 were excluded prior to downstream analysis.

After quality control, a total of 16 samples – including healthy donors (HD), MM, and SMM – were retained for downstream analysis. Violin plots of QC metrics (Fig. 2) showed that most cells across all samples exhibited comparable distributions of gene count (nFeature_RNA), unique molecular identifier (UMI) count (nCount_RNA), and mitochondrial gene content (percent.mt).

Specifically, the majority of cells presented nFeature_RNA values between 1000 and 6000, with relatively consistent profiles across conditions. nCount_RNA distributions were also centered in the 10 000–40 000 range, though slight right skewing was observed in some MM samples (e.g., MM2 and MM3), likely reflecting elevated transcript capture. Mitochondrial content (percent.mt) remained below 25% in most cells, indicating minimal stress or apoptosis during library preparation. These patterns confirmed the overall high quality and technical consistency of the single-cell transcriptomic data used in this study.

### Principal component analysis (PCA), cell clustering, and dimensionality reduction visualization

**Figure 3.**
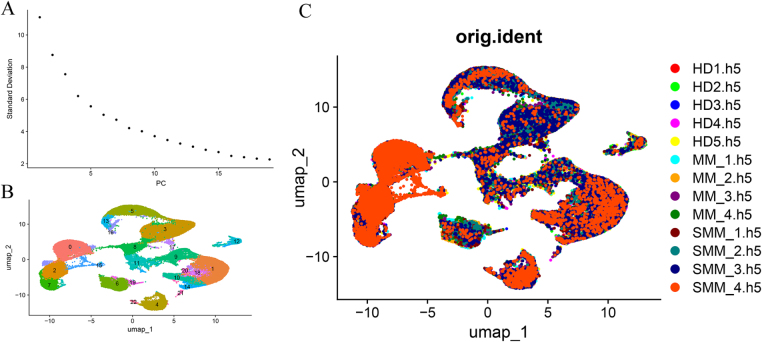
Cell clustering and sample grouping. (A) Principal component analysis (PCA) showing the variance explained by each principal component, guiding the dimensionality reduction process. (B) Uniform manifold approximation and projection (UMAP) plot showing distinct cell clusters identified through unsupervised clustering, labeled with cluster IDs. (C) UMAP plot displaying cells grouped by their sample origins, enabling comparisons between different experimental conditions.

The standard deviation curve (shown in Fig. 3A) indicated that the first 10 principal components (PCs) exhibited significantly higher values than the others, suggesting that these components capture the major sources of variation within the dataset. This result validated the use of the first 10 PCs for further analyses, providing a solid foundation for subsequent dimensionality reduction and clustering analyses.

Following PCA, UMAP (Uniform Manifold Approximation and Projection) was applied for dimensionality reduction. UMAP projects the high-dimensional data into a two-dimensional space, allowing for effective visualization of the cell populations. The UMAP plot (Fig. 3B) clearly showed distinct subgroups of cells, with excellent separation between clusters. This clear separation indicates that the UMAP analysis successfully captured the cellular diversity in the dataset, providing an intuitive way to visualize the cellular heterogeneity in the multiple myeloma samples.

Further analysis of the sample origin revealed that cells from different samples were evenly distributed across the clusters, as shown in Fig. 3C. This observation suggests that the clustering patterns were not biased by sample origin, indicating that the cell populations identified in the analysis reflect true biological variation rather than sample-specific effects. This robust clustering of cells sets the stage for more detailed functional and molecular investigations, enabling the identification of specific cell types and potential biomarkers for multiple myeloma.

### Cell annotation

**Figure 4.**
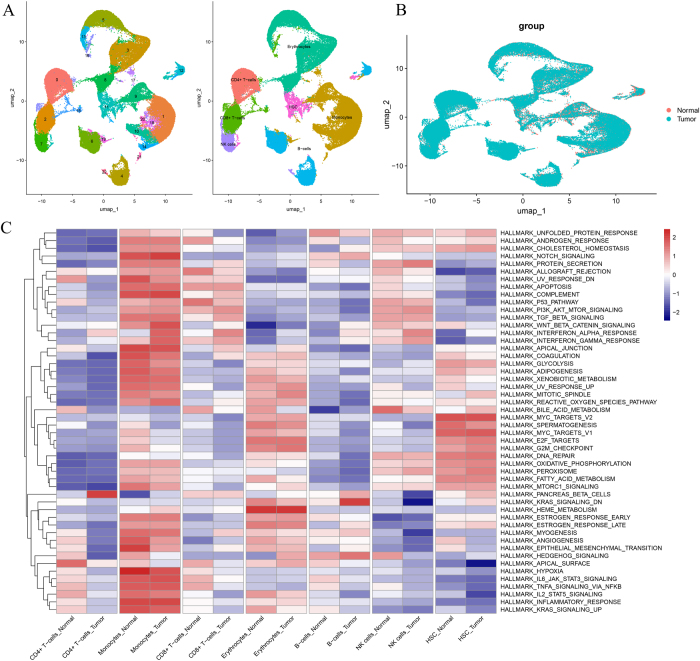
Cell type annotation and pathway analysis. (A) UMAP-based cell annotation using canonical marker genes, with distinct cell types, including T cells, monocytes, and NK cells, highlighted. (B) UMAP projection showing transcriptional clustering of cells derived from tumor and normal tissues. The plot reflects differences in gene expression patterns rather than physical spatial distribution, highlighting transcriptionally distinct cell populations. (C) Heatmap of gene set variation analysis (GSVA), illustrating differential pathway enrichment across tumor and normal cell types. Key pathways include immune response and tumor microenvironment remodeling.

By comparing the expression data of our cells with these established reference profiles, we were able to annotate a wide range of major cell types, including erythrocytes, CD4+ T cells, CD8+ T cells, monocytes, NK cells, B cells, and HSCs, as shown in Fig. 4A. However, this study mainly focuses on CD4+ T cells, CD8+ T cells, monocytes, NK cells, and B cells. Although erythrocytes and HSCs are included in subsequent results, they are not the primary focus and do not affect the interpretation of other findings.

Additionally, our analysis revealed transcriptional heterogeneity between cells derived from tumor and normal tissues. The UMAP plot (Fig. 4B) demonstrates that these cells form distinct clusters based on gene expression profiles. This clustering pattern indicates that tumor cells display transcriptional programs that are distinguishable from those of normal cells, rather than reflecting physical spatial separation. The distinct positioning of these cell clusters further implies that there may be differences in cell states and interactions between the two tissue types, contributing to the pathology of the tumor.

Figure 4 provides a detailed view of the cellular heterogeneity and spatial distribution within the tumor and normal tissue samples, supporting the hypothesis that tumor microenvironments are characterized by distinct transcriptional changes compared to normal tissue counterparts. These findings open new avenues for investigating the molecular mechanisms driving tumor progression and immune evasion in multiple myeloma and similar cancers.

### Screening of marker genes and analysis of GSVA pathways

Using GSVA, we evaluated the activity of signaling pathways across different cell types from tumor and normal samples. The GSVA heatmap (Fig. 4C) presents the activity levels of various pathways in the cell types from both groups, providing insights into the biological processes active in the tumor microenvironment.

The results revealed notable differences between the tumor and normal samples:

1. Tumor Samples: CD4+ T cells, CD8+ T cells, and monocytes in tumor samples showed significant upregulation of key tumor-related pathways, including the “PI3K-AKT-mTOR signaling pathway” and the “WNT-β-catenin signaling pathway.” These pathways are known to play crucial roles in promoting cell survival, proliferation, and tumor progression. Their upregulation in immune cells, such as T cells and monocytes, suggests that these cells might be actively involved in remodeling the tumor microenvironment, potentially contributing to immune evasion and tumor progression. In addition, tumor-related key signaling pathways such as the “TGF-β signaling pathway” and the “inflammatory response pathway” were also significantly upregulated across multiple cell types. This reflects the heightened inflammatory state within the tumor microenvironment, which often promotes tumor growth, angiogenesis, and immune suppression.

2. Normal Samples: In normal samples, erythrocytes and HSCs exhibited higher activity in the “oxidative phosphorylation” pathway. This increased activity may reflect the essential role of these cell types in homeostatic regulation and energy production under normal conditions. Erythrocytes, which are involved in oxygen transport, and HSCs, which contribute to hematopoiesis, likely require efficient energy metabolism to maintain their functions.

The GSVA heatmap (Fig. 4C) also indicates that various other signaling pathways, including the “MYC targets V2” and “Notch signaling,” are active in both tumor and normal conditions but exhibit significant changes in activity between the two tissue types. These findings suggest that tumor cells activate distinct sets of pathways to drive malignancy, while normal cells maintain metabolic and homeostatic functions.

**Figure 5.**
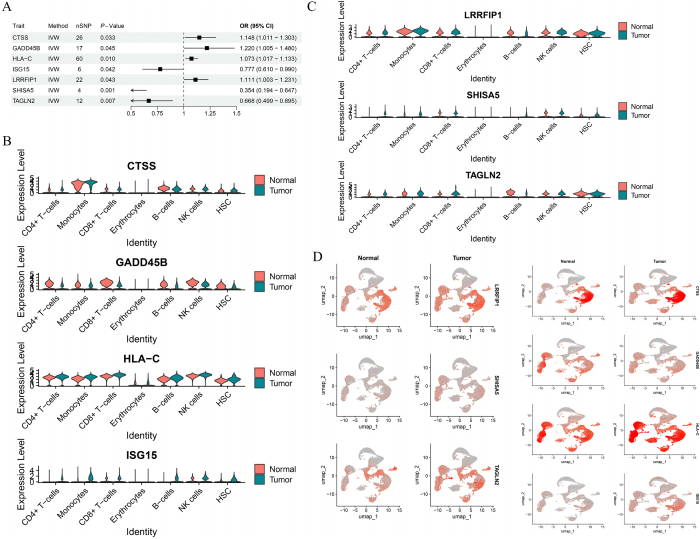
Gene expression and Mendelian randomization analysis. (A) EQTL analysis results for seven candidate genes linked to multiple myeloma, validated through MR. (B–C) Violin plots of gene expression levels for key genes (e.g., ISG15, HLA-C, and TAGLN2) in different immune cell types from tumor and normal samples. (D) UMAP plots showing spatial expression patterns of these genes across cell clusters, indicating their functional roles in tumor progression and immune modulation.

Moreover, the GSVA results (Fig. 4C) indicated robust enrichment of PI3K-AKT-mTOR and Wnt/β-catenin signaling specifically in CD4⁺ and CD8⁺ T cells from MM patients. Mechanistically, this aberrant activation may reflect immune exhaustion and regulatory skewing. Several MR-prioritized genes involved in these pathways – such as CTSS (linked to antigen processing and T cell differentiation via the PI3K axis) and LRRFIP1 (which modulates β-catenin stability in inflammatory environments) – were found to be significantly upregulated in these T cell subsets (Fig. 5C-D). These gene-specific findings support that oncogenic signaling in T cells may not be secondary to tumor presence but is genetically driven, contributing to a dysfunctional immune milieu in MM.

### Intersection genes and Mendelian randomization study of multiple myeloma

By intersecting the differential genes from immune cells, we identified 50 significant differential genes associated with multiple myeloma (Supplementary Digital Content Material 1, available at: http://links.lww.com/JS9/E990). Using eQTL data from these 50 genes, we conducted a two-sample MR study to explore the potential causal relationships between these genes and multiple myeloma.

The MR results indicated a positive causal relationship between HLA-C [OR = 1.073 (1.017–1.133), *P* = 0.010], LRRFIP1 [OR = 1.111 (1.003–1.231), *P* = 0.043], CTSS [OR = 1.148 (1.011–1.303), *P* = 0.033], and GADD45B [OR = 1.220 (1.005–1.480), *P* = 0.045] with multiple myeloma, suggesting that these genes may promote tumorigenesis or contribute to the progression of multiple myeloma. On the other hand, genes such as SHISA5 [OR = 0.354 (0.194–0.647), *P* = 0.001], TAGLN2 [OR = 0.668 (0.499–0.895), *P* = 0.007], and ISG15 [OR = 0.777 (0.610–0.990), *P* = 0.042] exhibited a negative causal relationship, indicating that these genes may play protective or suppressive roles against tumor development (Fig. 5A, Supplementary Digital Content Material 2, available at: http://links.lww.com/JS9/E991). To assess the robustness of the Mendelian randomization results and identify potential pleiotropic effects, we conducted multiple sensitivity analyses. MR-Egger regression and MR-PRESSO analyses revealed no evidence of horizontal pleiotropy among the instrumental variables used (Supplementary Digital Content Material 3, available at: http://links.lww.com/JS9/E992). Additionally, leave-one-out analysis demonstrated that the exclusion of any single SNP did not substantially alter the overall causal estimates between the selected eQTLs and multiple myeloma, indicating that the results were not driven by any single instrumental variable (Supplementary Digital Content Figure 1, available at: http://links.lww.com/JS9/E989). These findings collectively support the validity and robustness of our MR conclusions.

Further analysis was conducted to assess the differential expression of these genes between tumor and normal tissues. The violin plots (Fig. 5B-C) showed significant changes in gene expression, where CTSS, GADD45B, HLA-C, and LRRFIP1 were highly expressed in tumor samples, supporting their involvement in tumor progression. Conversely, SHISA5, TAGLN2, and ISG15 exhibited lower expression levels in the tumor samples, consistent with their role in suppressing the disease.

The UMAP plots (Fig. 5D) illustrated the spatial distribution of these genes across the tumor and normal tissues, revealing clear transcriptional differences between the two groups. The genes with a positive causal relationship (e.g., HLA-C, LRRFIP1) were more strongly expressed in tumor samples, while genes with a negative causal relationship (e.g., SHISA5, TAGLN2) showed higher expression in normal tissues.

These results suggest that these differential genes could be key players in the development and progression of multiple myeloma, providing new insights into potential biomarkers and therapeutic targets. Supplementary Digital Content Material 2, available at: http://links.lww.com/JS9/E991 further supports these findings, offering a detailed breakdown of the Mendelian randomization analysis and gene associations.

### Integrated analysis reveals cell-type-specific genetic pathways driving MM progression

To address the limitations of traditional bulk tissue studies in resolving the complexity of the MM microenvironment, we integrated eQTL-based MR with single-cell transcriptomic data. This approach allowed us to map genetically regulated gene expression onto specific immune cell populations, providing a high-resolution view of causal pathways in MM.

Specifically, GSVA analysis revealed that the PI3K-AKT-mTOR and WNT-β-catenin signaling pathways were significantly enriched in CD4⁺ and CD8⁺ T cells and monocytes from tumor samples compared to their counterparts in normal tissues (Fig. 4C). These pathways, classically implicated in tumor cell proliferation, demonstrated here to be upregulated in immune cells, suggesting that genetically regulated activation of these pathways may contribute to immune cell dysfunction and tumor-supportive phenotypes. This finding contrasts with previous bulk tissue studies where these pathways were largely attributed to malignant plasma cells, and underscores the importance of cellular resolution in attributing pathway activity to functionally distinct cell populations.

Furthermore, MR analysis identified several genes with significant causal associations with MM, including HLA-C, CTSS, and LRRFIP1 (positively associated) and SHISA5, TAGLN2, and ISG15 (negatively associated) (Fig. 5A). When mapped back onto the single-cell landscape, we observed that pro-tumor genes such as HLA-C and CTSS were predominantly expressed in CD4⁺ T cells and monocytes, while protective genes like ISG15 and TAGLN2 showed higher expression in NK cells and CD8⁺ T cells (Fig. 5B–D). This spatial resolution highlights how genetically regulated expression aligns with functional immune compartments, supporting the interpretation of gene-disease causality within the tumor-immune interface.

By combining MR with single-cell mapping, we not only identified candidate genes with causal roles in MM but also linked these genes to the specific immune cells through which they may exert their effects, thereby enhancing the biological interpretability and clinical relevance of the findings. For example, ISG15, a protective gene downregulated in MM (Fig. 5B), was further prioritized for small molecule screening due to its distinct expression in CD8⁺ T cells – implicating a potential role in cytotoxic T cell activation and immune reprogramming.

### Cell communication patterns and ligand–receptor pairs

**Figure 6.**
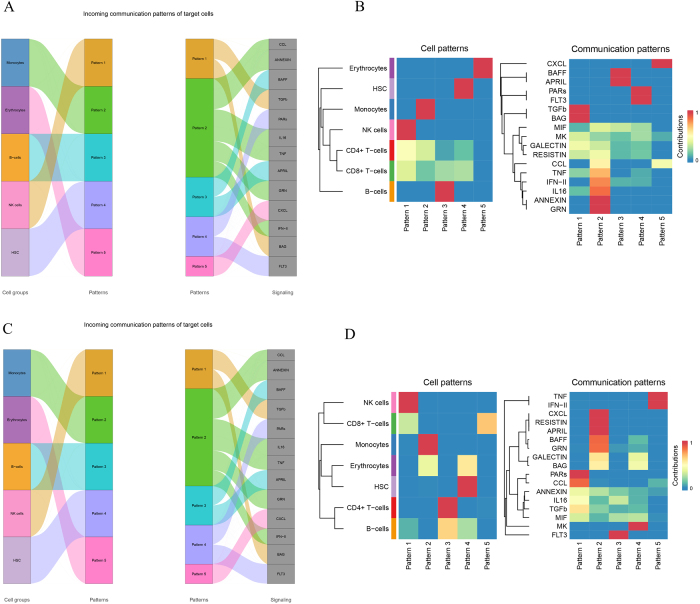
Cellular communication patterns and signal allocation. (A–B) Sankey diagrams visualizing incoming and outgoing communication patterns among cell types. Key signaling pathways, including cytokine and chemokine-mediated signaling, are highlighted. (C–D) Heatmaps showing the contribution of specific signaling molecules (e.g., CXCL, IL16, and BAFF) in mediating intercellular communication.

In the inbound communication pattern analysis, five significant patterns (Patterns 1–5) were identified, involving a variety of cell types such as erythrocytes, HSCs, monocytes, NK cells, CD4+ T cells, CD8+ T cells, and B cells (Fig. 6A-B). The main signaling pathways in these patterns included CXCL, BAFF, and TGF-β. Notably, NK cells played a prominent role in Pattern 3, while B cells were more actively involved in Patterns 4 and 5. This indicates distinct functional roles for different cell types in regulating inbound communication networks.

For the outbound communication pattern analysis, five significant communication patterns were also observed (Fig. 6C-D). NK cells, CD8+ T cells, and monocytes were the primary signal senders, with specific signaling molecules such as TNF, IFN-II, and GRN playing key roles in Pattern 2. Additionally, certain molecules like annexin and resistin were highly associated with specific cell types, such as CD4+ T cells in Pattern 5 and monocytes in Patterns 1 and 3.

**Figure 7.**
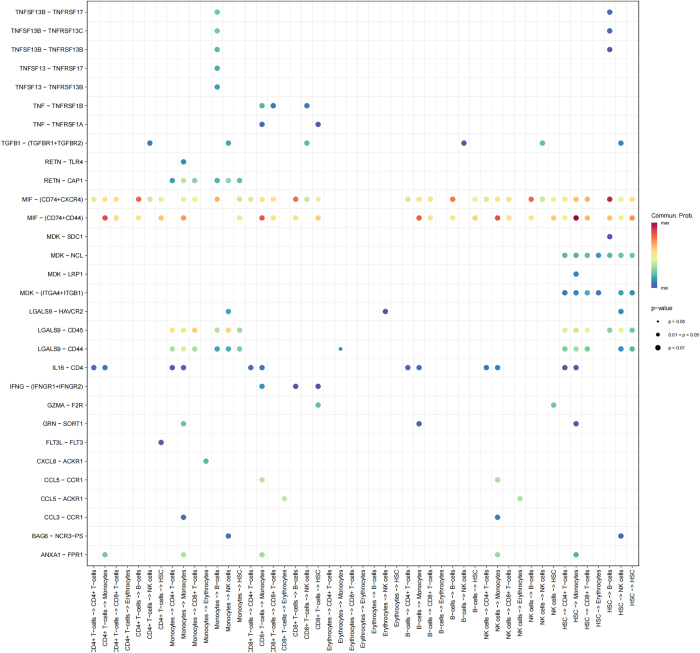
Comprehensive receptor–ligand interaction networks. A comprehensive representation of receptor–ligand interaction pairs identified in the tumor microenvironment. This figure highlights the dominant signaling interactions mediating cell–cell communication, including pathways involved in immune regulation and tumor progression.

Further analysis of ligand–receptor pair interactions revealed crucial communication mechanisms between cell types (Fig. 7). For instance, CD4+ T cells and monocytes predominantly communicated via the “CCL5-CCR1” and “IL16-CD4” pathways, while CD8+ T cells interacted with B cells and NK cells through the “TNF-TNFRSF1A” and “CXCL8-ACKR1” pathways. Communication within erythrocytes was mediated primarily by the “GRN-SORT1” pathway, and HSCs regulated interactions with other cell types via the “FLT3L-FLT3” pathway.

These results shed light on the specific cell interactions and signaling pathways involved in tumor progression, offering insights into the complex immune network regulating multiple myeloma and its microenvironment.

### The functional roles of major communication networks

**Figure 8.**
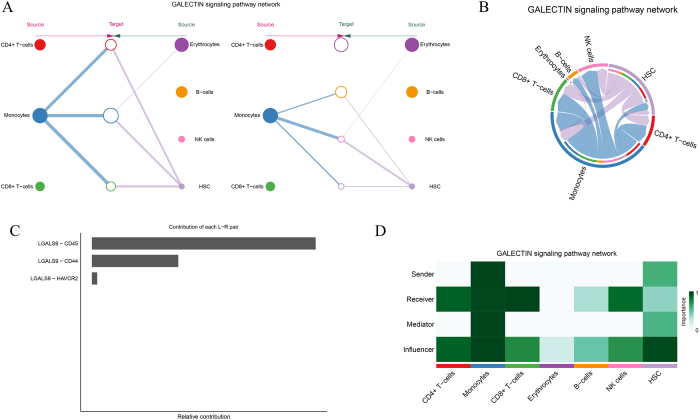
GALECTIN signaling pathway analysis. (A–B) Interaction networks of the GALECTIN pathway across cell types, focusing on CD4+ T cells, monocytes, and NK cells. (C) Quantification of receptor–ligand pairs (e.g., LGALS9-CD44, LGALS9-HAVCR2) contributing to pathway activity. (D) Heatmap summarizing the roles of cell types as senders, receivers, mediators, or influencers in GALECTIN signaling, emphasizing its central role in immune suppression.

The GALECTIN signaling network plays a critical role in regulating various immune cell types, including CD4+ T cells, CD8+ T cells, monocytes, erythrocytes, B cells, NK cells, and HSCs (Fig. 8A-B). The GALECTIN signaling pathway is particularly active in immune-related cells such as T cells and monocytes, with erythrocytes and HSCs contributing to signal transmission and reception, albeit to a lesser extent (Fig. 8C). Ligand–receptor pair analysis further highlighted the importance of specific interactions, with key pairs like LGALS9-HAVCR2, LGALS9-CD44, and LGALS9-CD45 playing central roles in the signal transmission (Fig. 8D). These findings underscore the diverse and complex nature of the GALECTIN signaling network in regulating immune responses, especially within the tumor microenvironment.

**Figure 9.**
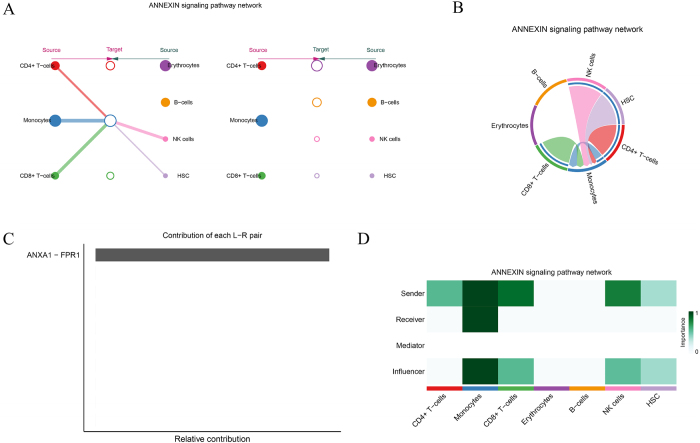
ANNEXIN signaling pathway analysis. (A–B) Signaling network analysis of the ANNEXIN pathway, highlighting interactions between CD4+ T cells, monocytes, and NK cells. (C) Dominant receptor–ligand pair ANXA1-FPR1 identified as a key mediator of this pathway. (D) Functional heatmap categorizing cell roles in ANNEXIN signaling, revealing their contributions to intercellular communication and inflammation.

The ANNEXIN signaling network is also widely present across various immune cell types, including CD4+ T cells, CD8+ T cells, monocytes, erythrocytes, and NK cells, where it modulates intercellular immune signaling (Fig. 9A-B). In this network, monocytes, CD4+ T cells, and CD8+ T cells are the primary signal senders, transmitting signals through the core ligand–receptor pair ANXA1-FPR1, which plays a crucial role in immune modulation (Fig. 9C). The ANXA1-FPR1 interaction appears to contribute significantly more than other ligand–receptor pairs, indicating its central role in the immune regulation within the tumor microenvironment. Additionally, ANNEXIN signaling analysis revealed the intricate transmission or reception of signals between different cell types (Fig. 9D), offering a foundation for future studies on its role in immune regulation.

**Figure 10.**
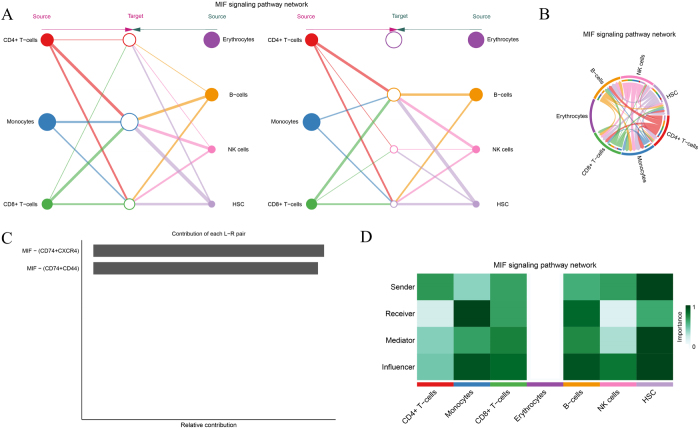
MIF signaling pathway network. (A–B) Visualization of the MIF signaling network, showing interaction patterns across immune and stromal cells. (C) Quantitative analysis of receptor–ligand pairs (e.g., MIF-CD74, MIF-SDC1) mediating tumor-immune interactions. (D) Functional categorization of cells based on their roles (sender, receiver, mediator, or influencer) in the MIF pathway, with implications for immune evasion mechanisms.

The MIF (Macrophage Migration Inhibitory Factor) signaling network is vital for immune regulation in CD4+ T cells, CD8+ T cells, monocytes, erythrocytes, B cells, and NK cells (Fig. 10A). This pathway demonstrates significant immune regulatory capabilities within the tumor microenvironment. In the MIF signaling network, CD4+ T cells and monocytes primarily act as the signal secretors, while HSCs, B cells, and NK cells serve as the major signal recipients, suggesting their roles as key effector cells downstream of MIF signaling (Fig. 10B). Ligand–receptor pair analysis of MIF signaling uncovered complex molecular interactions between cells (Fig. 10C). Different cell types in the signaling network function as senders, receivers, mediators, or influencers, emphasizing the diverse and critical role of MIF signaling in immune regulation (Fig. 10D).

These findings highlight the intricate and diverse roles of GALECTIN, ANNEXIN, and MIF signaling pathways in the tumor microenvironment and their potential as therapeutic targets for modulating immune responses in diseases like multiple myeloma. By mapping the specific cell types involved and their ligand–receptor interactions, we gain a deeper understanding of the complex immune landscape that contributes to tumor progression and immune evasion.

### Molecular docking

Using CTD, we identified small molecules that may influence the predicted proteins associated with multiple myeloma (Supplementary Material 4, available at: http://links.lww.com/JS9/E993). After reviewing the compounds, we decided not to further analyze GADD45B, as it has already been extensively studied in previous research. Additionally, no small molecules targeting SHISA5 were identified in our search, and therefore, this gene was excluded from further investigation.

**Figure 11.**
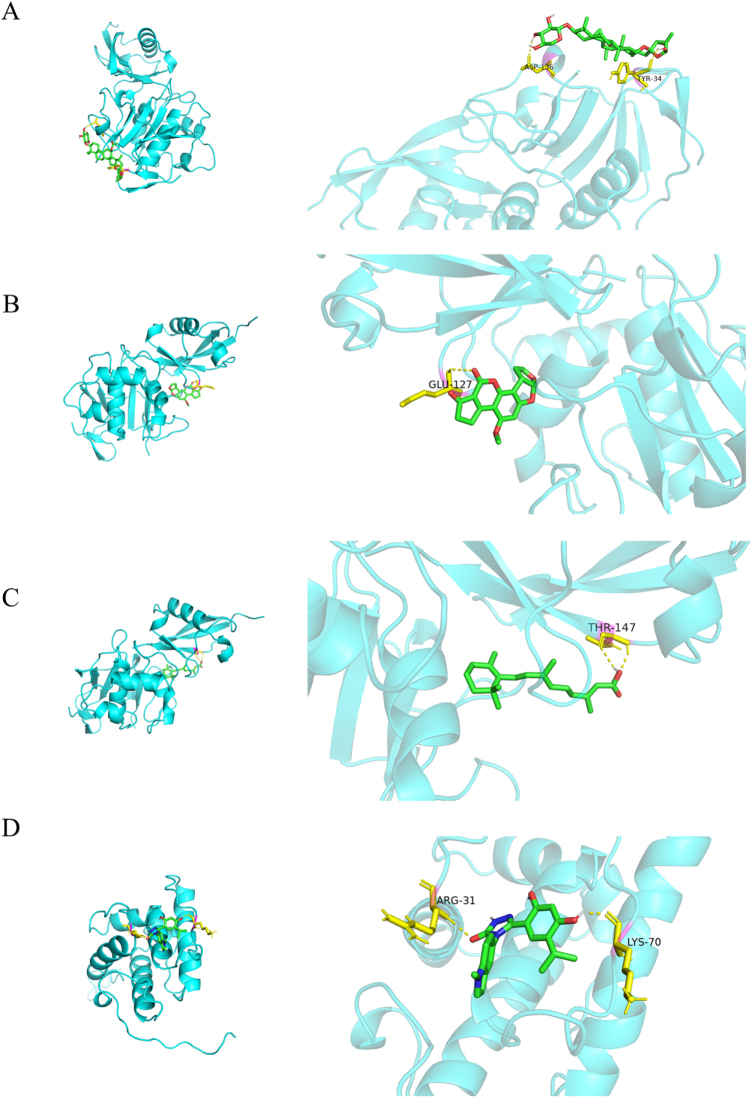
Molecular docking results for candidate therapeutics. (A–D) Binding interactions between key therapeutic compounds (e.g., actein, aflatoxin B1, tretinoin, STA 9090) and candidate proteins (e.g., ISG15 and TAGLN2). Binding energies and docking scores demonstrate strong affinity, supporting their potential as therapeutic agents targeting immune-related pathways in multiple myeloma.

After analyzing the docking results, we found that several small molecules showed promising interactions with the target proteins, meeting the docking score condition of <−5, indicating a strong binding affinity. Specifically, ISG15 showed promising docking interactions with actein, aflatoxin B1, and tretinoin, while TAGLN2 demonstrated significant interaction with STA 9090 (Fig. 11, Supplementary Material 4, available at: http://links.lww.com/JS9/E993). These results suggest that these compounds may be potential modulators of the target proteins, offering new avenues for therapeutic development in treating multiple myeloma.

Figure 11 provides visual evidence of the docking interactions, showing the molecular binding between the small molecules and their target proteins, which further supports ISG15 and TAGLN2 as potential drug targets. The docking scores for these interactions indicate strong binding, which could be crucial for the development of novel therapies aimed at regulating these proteins in the context of cancer.

## Discussion

MM is driven by complex interactions between tumor and immune cells within the bone marrow microenvironment. Our findings provide new insights into how immune dysregulation, ligand–receptor signaling, and genetically regulated pathways contribute to disease progression and therapy resistance.

MM progression is closely linked to immune evasion and clonal evolution within a permissive bone marrow microenvironment. Despite advances in therapy, most patients eventually relapse due to drug resistance, emphasizing the need to uncover regulatory mechanisms that sustain tumor survival and immune suppression^[46,47]^. Beyond clonal evolution, recent studies have shown that the bone marrow microenvironment actively promotes drug resistance through signaling molecules, cell adhesion, and immune suppression^[48,49]^. Our findings suggest that upregulated pathways such as PI3K-AKT-mTOR and TGF-β not only drive proliferation but may also mediate cell survival signals that undermine therapy. Further experimental validation is needed to determine whether these pathways directly support the persistence of drug-resistant subclones, and whether targeting microenvironment-induced resistance can enhance therapeutic responses. This study aimed to explore the cellular characteristics, immune interactions, and signaling pathways that drive disease progression, leveraging scRNA-seq.

We found that immune cells, particularly CD4+ T cells, CD8+ T cells, NK cells, and monocytes, exhibit altered gene expression within the tumor microenvironment compared to normal tissues. These immune cells frequently fail to launch an effective immune response, which has long been implicated in the phenomenon of immune evasion in MM[50]. Specifically, CD4+ and CD8+ T cells, essential for anti-tumor immunity, displayed heightened inflammatory and survival signaling pathways, such as the PI3K-AKT-mTOR and WNT-β-catenin pathways[51]. These pathways are well-established for their roles in promoting cell survival, proliferation, and immune evasion. Their activation in immune cells may not only drive tumor growth but also contribute to immune suppression, as these cells are reprogrammed to support tumor progression rather than combat it[52]. These observations are supported by our GSVA-based single-cell transcriptomic analysis (Fig. 4C), which revealed consistent upregulation of PI3K-AKT-mTOR and Wnt/β-catenin pathways in CD4⁺ and CD8⁺ T cells from MM samples compared to controls. This pathway activation pattern suggests not only a transcriptional reprogramming of immune cells, but also a genetically influenced remodeling of T cell signaling. Recent studies have shown that PI3K-AKT signaling in CD8⁺ T cells promotes terminal exhaustion and loss of effector function, contributing to immune evasion in hematological malignancies[53]. Similarly, Wnt/β-catenin pathway activation in CD4⁺ T cells skews differentiation toward immunosuppressive Treg phenotypes, further weakening anti-tumor immunity[54].

Compared to conventional therapies that primarily target malignant plasma cells, redirecting therapeutic focus toward genetically dysregulated immune cells may offer unique advantages. Specifically, targeting the PI3K or Wnt signaling in dysfunctional T cells could restore cytotoxicity, reduce immune exhaustion, and enhance the efficacy of checkpoint blockade or CAR-T cell therapies. This cell-type-specific approach complements tumor-directed strategies by remodeling the immune ecosystem, thereby providing a dual-pronged therapeutic benefit. This observation aligns with previous studies that have underscored the immune system’s failure to recognize and respond effectively to tumor cells in MM[55].

These findings suggest that targeting the PI3K-AKT-mTOR and WNT-β-catenin pathways in immune cells could be a viable therapeutic strategy to enhance immune responses and overcome immune evasion in MM. Additionally, the upregulation of inflammatory pathways, including TGF-β and NF-κB signaling cascades, further implicates the immune microenvironment in promoting MM progression. These pathways are central drivers of immune suppression, tumor growth, and drug resistance across various cancers, including MM[56]. In particular, the TGF-β pathway has been linked to tumor-associated inflammation and fibrosis in the bone marrow microenvironment of MM patients[57]. Our study suggests that targeting these immune-modulatory pathways could potentially restore immune activity against myeloma cells and enhance the effectiveness of current therapies, such as immune checkpoint inhibitors, that aim to re-establish immune surveillance.

The MR analysis conducted in this study provided pivotal insights into the genetic foundations of MM, allowing us to draw causal links between genetic variants and gene expression levels. By integrating eQTL data, we identified several genes, such as HLA-C, LRRFIP1, CTSS, and GADD45B, that showed a positive causal association with MM. Among these, the identification of HLA-C as a key genetic driver is particularly significant. HLA-C, a vital component of the major histocompatibility complex (MHC) class I molecule, plays a central role in antigen presentation and immune surveillance[58]. The increased expression of HLA-C in MM cells may facilitate immune evasion, as these cells can alter the expression of MHC molecules to evade detection by the immune system. The positive causal relationship between HLA-C and MM reinforces the hypothesis that HLA-C overexpression could be a critical factor in immune escape and suggests it as a promising therapeutic target[59].

Further analysis of the MR results revealed that LRRFIP1 and CTSS are strongly associated with MM progression. LRRFIP1, a gene involved in immune regulation, has been implicated in various immune-related processes, including the modulation of Toll-like receptor (TLR) signaling[60]. The causal link between LRRFIP1 and MM may indicate its role in promoting immune suppression within the tumor microenvironment, positioning it as an attractive target for therapies aimed at enhancing immune responses. Similarly, CTSS, a lysosomal protease, has been linked to tumor progression across multiple malignancies, including MM[61]. Our study suggests that CTSS may contribute to tumor progression and immune evasion by modulating the immune environment and degrading extracellular matrix components, thereby facilitating myeloma cell migration and invasion.

Interestingly, we observed a negative causal relationship between MM and genes such as SHISA5, TAGLN2, and ISG15, pointing to their potential as tumor suppressors. SHISA5 and TAGLN2 have been implicated in cellular processes such as migration and adhesion, and their downregulation in MM could enhance tumor cell motility and invasiveness[62]. The negative correlation of these genes with MM suggests that upregulating them could have therapeutic potential by inhibiting tumor progression. ISG15, known for its involvement in the antiviral immune response, has also been linked to cellular stress responses and immune modulation[63]. The inverse association between ISG15 expression and MM suggests that boosting its expression could strengthen anti-tumor immunity, making ISG15 a promising candidate for immunotherapy.

A major strength of our study is the in-depth analysis of cellular communication within the tumor microenvironment, enabled by tools such as CellChat and CellPhoneDB. Our investigation revealed significant differences in the ligand–receptor interactions between immune and stromal cells in the MM microenvironment. These findings highlight that tumor-immune-stromal crosstalk is not static, but undergoes dynamic remodeling during MM progression. The reprogramming of immune cells by stromal and tumor-derived ligands, as seen in the ANNEXIN and GALECTIN networks, suggests a bidirectional communication system that actively shapes tumor evolution. Future work incorporating spatial transcriptomics could further clarify how physical proximity and niche-specific signaling influence these interactions. Notably, we identified several key signaling pathways, including GALECTIN, ANNEXIN, and MIF, as major drivers of immune modulation and tumor progression.

The GALECTIN signaling pathway, which regulates immune responses and cell interactions, was found to be particularly active in T cells and monocytes, two critical immune populations in MM pathogenesis. The involvement of GALECTIN signaling in immune suppression is consistent with its role in various cancers, where it helps tumors evade immune detection by modulating T cell and dendritic cell function[64]. Targeting GALECTIN signaling in MM could provide a strategy for restoring immune responses and improving the effectiveness of immunotherapies.

Similarly, the ANNEXIN signaling pathway, involved in intercellular communication and inflammation, was identified as a key mediator of immune responses in the MM microenvironment. Our analysis pinpointed the ANXA1-FPR1 ligand–receptor pair, which regulates immune cell trafficking and function[65]. Activation of this pathway in MM suggests it may facilitate the recruitment of immune cells to the tumor microenvironment, where they are reprogrammed to support tumor growth rather than attacking the myeloma cells. Targeting the ANNEXIN pathway could disrupt this immune modulation and restore immune function.

The MIF signaling pathway was also identified as a critical mediator of immune evasion in MM. MIF, a pro-inflammatory cytokine, plays a role in immune regulation, and its upregulation in MM has been linked to tumor progression and immune suppression[66]. Identifying MIF as a key signaling node in MM offers a potential therapeutic target. By targeting the MIF pathway, it may be possible to enhance immune responses and inhibit tumor growth, providing a new approach for treating patients with relapsed or refractory MM.

In addition to genetic and cellular analyses, we employed molecular docking simulations to identify small molecules capable of modulating the activity of key proteins involved in MM progression. The docking results revealed strong binding interactions between these small molecules and proteins such as ISG15 and TAGLN2, suggesting their potential as therapeutic targets. Notably, ISG15 demonstrated promising interactions with several small molecules, including actein and aflatoxin B1, which may help regulate the immune response and inhibit tumor progression^[67,68]^. In addition to their potential as therapeutic targets, ISG15 and TAGLN2 may also play functional roles in mediating drug resistance in MM. ISG15, a ubiquitin-like modifier, has been implicated in proteasome pathway regulation and stress response. In the context of MM, where proteasome inhibitors such as bortezomib represent frontline therapies, dysregulation of ISGylation may alter proteostasis and contribute to resistance by stabilizing anti-apoptotic proteins or modulating immune-related signaling pathways. Moreover, recent studies suggest that ISG15 overexpression can dampen interferon responses and promote tumor survival under chemotherapeutic pressure^[69,70]^. TAGLN2, on the other hand, is a cytoskeletal regulator involved in actin dynamics and has been associated with drug resistance in solid tumors by modulating cell motility, adhesion, and survival^[71,72]^. In MM, increased TAGLN2 expression may enhance stromal interactions or affect the intracellular trafficking of therapeutic agents, thereby impairing drug efficacy. Our identification of strong docking affinities between these proteins and specific small molecules provides a rationale for further investigating whether pharmacologic modulation of ISG15 or TAGLN2 can overcome microenvironment-mediated resistance and restore treatment sensitivity. These findings underscore the potential of small molecules to target the tumor microenvironment and enhance the effectiveness of existing therapies.

The insights gained from this study have important clinical implications. The identification of key genetic drivers, immune pathways, and cellular interactions in MM opens up new possibilities for therapeutic intervention. Targeting the PI3K-AKT-mTOR and WNT-β-catenin pathways, for example, could provide effective strategies for overcoming immune evasion and boosting immune responses. Furthermore, the identification of promising small molecules that target key proteins such as ISG15 and TAGLN2 paves the way for the development of personalized therapies tailored to individual patient profiles. Combining molecular targeting strategies with existing immunotherapies also offers a promising approach to improving patient outcomes. However, further validation of these findings in preclinical and clinical settings will be essential to translate these discoveries into effective therapies. Although this study provides rich molecular insight, a key limitation is the absence of clinical predictive models. Integrating our identified genetic markers and pathway activity scores into prognostic models – such as immune cell-derived risk scores or ML-based classifiers – could enhance early risk stratification and guide therapy selection. Future work will focus on leveraging large-scale clinical datasets and AI-driven modeling to translate these molecular findings into actionable decision-support tools for MM management. In conclusion, this study provides a comprehensive and detailed analysis of the genetic, cellular, and molecular mechanisms underlying MM. By identifying critical signaling pathways, cellular interactions, and genetic drivers, it lays the foundation for developing innovative therapeutic strategies aimed at improving the prognosis for patients suffering from this complex malignancy.

## Data Availability

None.
